# Genetic and non-genetic factors influencing phenotypic variability in neurofibromatosis type 1

**DOI:** 10.1186/s13023-026-04334-1

**Published:** 2026-04-15

**Authors:** Patricia Bianca Clissa, Sabri Saeed Sanabani

**Affiliations:** 1 Immunopathology Laboratory, Butantan Institute, Avenida Vital Brasil, 1500, Sao Paulo, SP 05503-900 Brazil; 2 Laboratory of Medical Investigation LIM-56/03, Division of Dermatology, Faculty of Medicine, University of Sao Paulo, Sao Paulo, 05403 000 Brazil

**Keywords:** Neurofibromatosis type 1, Neurofibromin, Phenotypic heterogeneity, Genetic modifiers, Ras signaling pathway

## Abstract

Neurofibromatosis Type 1 (NF1), an autosomal dominant genetic disorder, is characterized by extensive clinical variability, posing significant challenges for prognosis and patient management. Despite being caused by mutations in a single gene, *NF1*, the expressivity of the disease ranges from mild cutaneous manifestations to severe, life-threatening complications, including malignant tumors, skeletal deformities, and cognitive impairments. This paper provides a comprehensive review of the genetic determinants underlying this phenotypic heterogeneity. We begin by elucidating the molecular genetics of NF1, detailing the structure and function of the *NF1* gene and its protein product, neurofibromin, and the diverse spectrum of mutations that cause the disorder. Neurofibromin’s critical role as a negative regulator of the Ras signaling pathway is central to understanding the pathophysiology of NF1. Subsequently, the paper explores the primary mechanisms driving phenotypic variation, including established genotype-phenotype correlations, the influence of genetic modifiers, the impact of somatic mosaicism and second-hit mutations, and the emerging role of epigenetic and environmental factors. By synthesizing current knowledge, this review aims to construct a holistic view of the complex interplay between the primary *NF1* mutation and the broader genetic landscape that ultimately shapes the clinical presentation of NF1. Understanding these determinants is crucial for advancing diagnostic capabilities, developing personalized therapeutic strategies, and improving clinical outcomes for individuals affected by this complex disorder.

## Introduction

Neurofibromatosis Type 1 (NF1) is one of the most common single-gene disorders affecting the human nervous system, with a prevalence of approximately 1 in 3000 individuals worldwide [1], It is inherited in an autosomal dominant pattern, with about half of the cases arising from de novo mutations. The clinical diagnosis of NF1 is typically established based on criteria defined by the National Institutes of Health (NIH) Consensus Development Conference, which include characteristic features such as café-au-lait macules, skinfold freckling, cutaneous neurofibromas, Lisch nodules, optic pathway gliomas, and specific bone abnormalities [2]. Affected individuals also are at increased risk for hypertension, cardiovascular anomalies, and certain malignancies, making early diagnosis and screening a critical aspect of care [3].

Despite its monogenic origin, NF1 is renowned for its extreme clinical variability, a phenomenon that complicates every aspect of patient care, from genetic counseling to long-term management [4]. The phenotypic spectrum is remarkably broad, with manifestations varying in number, type, and severity not only between unrelated individuals but also among affected members of the same family who share the identical germline *NF1* mutation [5–7]. For instance, one individual may present with only mild dermatological signs, such as café-au-lait spots and a few cutaneous neurofibromas, leading a relatively unaffected life. In contrast, another individual, even a close relative, might develop severe complications such as large, disfiguring plexiform neurofibromas, which can undergo malignant transformation into malignant peripheral nerve sheath tumors (MPNSTs), aggressive optic gliomas leading to vision loss, or debilitating skeletal dysplasia. This profound heterogeneity presents a fundamental paradox: how can a defect in a single gene produce such a diverse array of clinical outcomes?

The resolution to this paradox lies in a complex interplay of genetic, epigenetic, and potentially environmental factors that modulate the expression of the primary *NF1* gene defect. While the germline mutation in the *NF1* gene is the initiating event, it does not act in a vacuum. The subsequent clinical course is shaped by a multifactorial landscape that includes the specific type of *NF1* mutation, the influence of other genes in an individual’s genome (modifier genes), stochastic events such as somatic “second-hit” mutations, and epigenetic modifications that regulate gene expression without altering the DNA sequence [8].

This article aims to review the current understanding of the genetic determinants that drive phenotypic variability in NF1. By exploring the molecular basis of the disorder, examining the mechanisms that generate clinical diversity, and linking these genetic factors to specific clinical manifestations, we seek to provide a comprehensive framework for appreciating the complexity of NF1 pathogenesis. A deeper understanding of these determinants is not merely an academic exercise; it holds the key to developing more accurate prognostic tools, identifying high-risk patients who require intensive surveillance, and designing targeted therapies that can be tailored to an individual’s unique genetic profile. Ultimately, unraveling the sources of phenotypic variability is essential for moving towards a new era of personalized medicine for individuals living with NF1.

## The molecular genetics of NF1

Understanding the profound phenotypic variability in NF1 begins with a detailed examination of its molecular basis: the NF1 gene, its protein product neurofibromin and the consequences of its dysfunction. The size of the gene, its complex structure and the multi-layered role of the protein in cellular signaling provide fertile ground for the emergence of clinical diversity.

### The *NF1* gene and neurofibromin protein

**Fig. 1:**
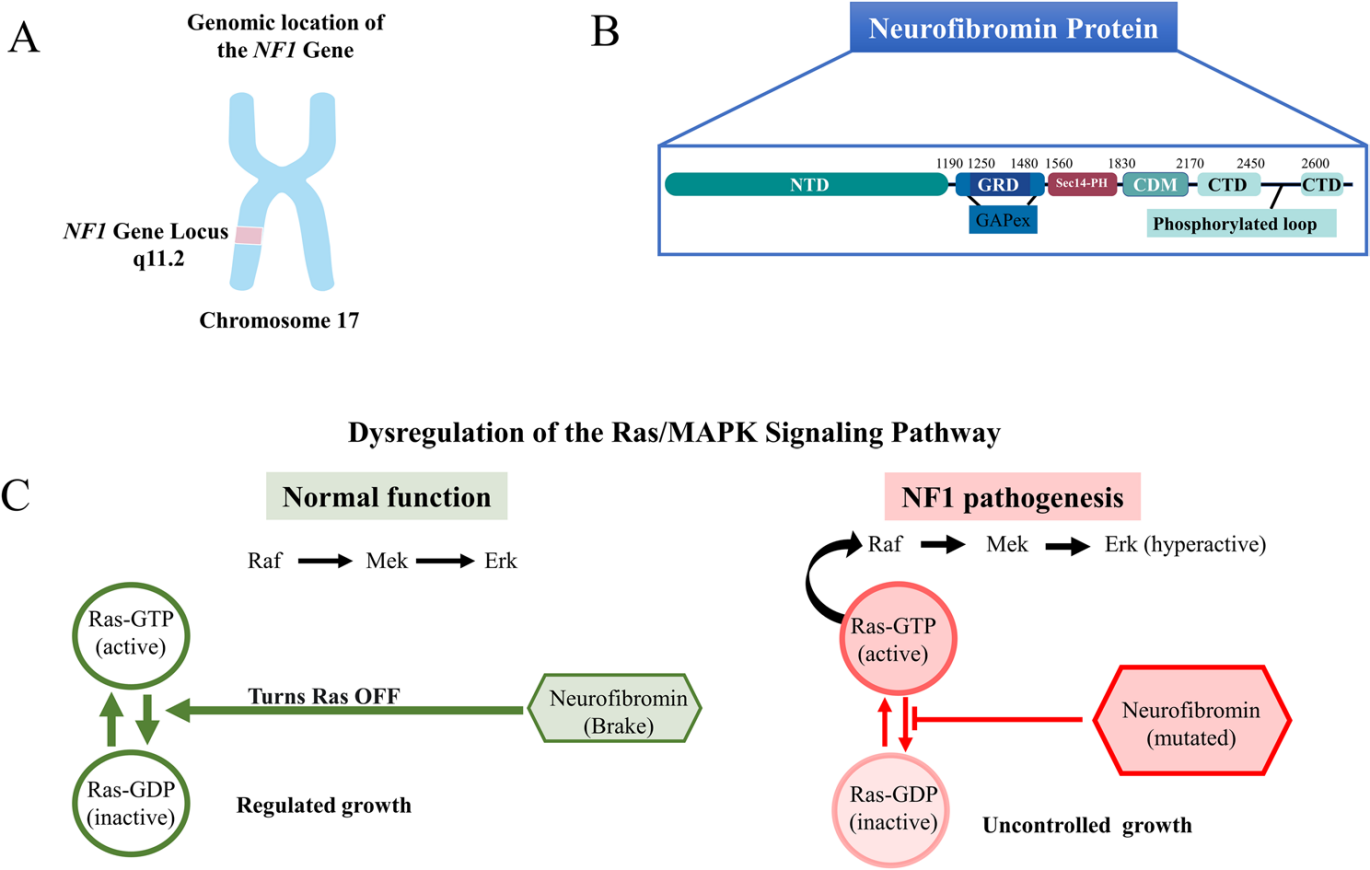
Genomic location, domain structure, and molecular basis of NF1 pathogenesis. (**A**) The NF1 gene is located on chromosome 17q11.2. (**B**) It encodes the neurofibromin protein, which contains multiple functional domains. (**C**) Neurofibromin negatively regulates the Ras pathway by promoting the conversion of active Ras-GTP to inactive Ras-GDP. In NF1 pathogenesis (right), mutated neurofibromin cannot perform this function, resulting in constitutive Ras activation and hyperactivation of the downstream Raf-MEK-ERK cascade, leading to uncontrolled tumor growth

The *NF1* gene, identified over a period of three years between 1987 and 1990, is one of the largest genes in the human genome, spanning over 280 kilobases (kb) of genomic DNA on chromosome 17q11.2 [9, 10]. Its considerable size and complex genomic organization, which includes 60 exons, contribute to its high spontaneous mutation rate, one of the highest for any known human disease gene [11, 12]. This high rate explains the significant proportion of NF1 cases (approximately 50%) that occur sporadically, without a family history of the disorder [13]. The gene’s coding sequence is transcribed into a large messenger RNA (mRNA) of about 11–13 kb, which is then translated into a protein called neurofibromin (Figs. 1A, 1B) [14].

Neurofibromin is expressed in many cell types, with particularly high levels in neurons, Schwann cells, oligodendrocytes, astrocytes, and leukocytes, and is most abundant in adult neuronal cells [15, 16]. This widespread expression pattern corresponds with the multisystemic nature of NF1, where dysfunction can affect the nervous system, skin, bone, and other tissues. Several NF1 transcript variants, generated by alternative splicing of pre-messenger RNA, have been identified, resulting in different neurofibromin isoforms.

These isoforms, produced by the inclusion or exclusion of specific exons, are expressed at different levels in various tissues and developmental stages. Although neurofibromin isoform I is the most common, other isoforms may have subtle functional differences, including altered abilities to regulate Ras. This differential expression and function of isoforms is another potential mechanism contributing to the tissue-specific manifestations and overall phenotypic variability of NF1. The most common NF1 mRNA form contains 57 exons and encodes neurofibromin isoform I, which consists of 2818 amino acids [16]. Neurofibromin is a multidomain, multifunctional giant protein composed of an N-terminal domain (NTD), GTPase-activating protein (GAP)-related domain (GRD), GAP-extra domain (GAPex), secretory protein 14-pleckstrin homology-like module (Sec14-PH), central dimerization module (CDM), and a C-terminal domain (CTD) interrupted by a loop with reported phosphorylation sites (Fig. 1B) [17]. The central and most well-characterized functional region of neurofibromin is its GRD, located in the middle of the protein [18]. This domain is structurally and functionally homologous to other GAPs, which are key regulators of the Ras family of small GTPases [19]. In addition to the GRD, neurofibromin contains several other domains, including a cysteine-serine-rich domain (CSRD), a tubulin-binding domain, and a C-terminal syndecan-binding domain, indicating its involvement in various cellular processes beyond Ras regulation (Fig. 1B) [16].

### Spectrum of *NF1* gene variants

The mutational landscape of the NF1 gene is highly diverse, with more than 3100 different variants identified to date [20]. Throughout this review, the classification of variants as “pathogenic” or “likely pathogenic” follows the evidence-based standards and guidelines established by the American College of Medical Genetics and Genomics (ACMG) and the Association for Molecular Pathology (AMP) [21]. These mutations are distributed throughout the entire length of the gene, with very few mutational hotspots. This allelic heterogeneity is a primary contributor to the clinical variability observed in the disorder. The mutations can be broadly categorized into several types:

1. **Truncating pathogenic variants:** These are the most common variants, collectively comprising nonsense and frameshift variants. Nonsense variants introduce a premature stop codon directly into the coding sequence, while frameshift variants (caused by insertions or deletions that are not a multiple of three) alter the reading frame, which almost always leads to a downstream premature stop codon [22]. In both cases, the ultimate consequence is the production of a truncated, unstable, and non-functional neurofibromin protein, leading to a state of haploinsufficiency.
2. **Splice-site pathogenic variants:** These variants occur at or near the junctions between exons and introns, disrupting the normal process of mRNA splicing. They are a very common cause of disease in NF1, accounting for a significant portion of pathogenic variants [23]. The functional consequences can be varied; while many splice-site variants cause the skipping of an entire exon, which frequently leads to a frameshift and premature truncation, others can result in in-frame deletions or the retention of intronic sequence. This variability in molecular outcome is another contributor to the overall phenotypic heterogeneity of the disorder.
3. **Missense pathogenic variants:** These variants, which involve the substitution of a single amino acid, account for about 10–17% of pathogenic NF1 variants and have heterogeneous functional consequences [13]. Pathogenic missense variants in NF1 are typically classified as loss-of-function (LoF), either by disrupting protein stability or impairing a specific function; true gain-of-function is not a primary disease mechanism. The phenotypic impact often depends on the affected domain. Variants within the critical GAP-related domain (GRD), for example, can severely impair neurofibromin’s catalytic ability to regulate Ras, effectively mimicking a complete loss of this key function [24, 25]. In contrast, missense variants in other domains may affect different aspects of neurofibromin function, such as its interactions with other proteins. This domain specificity helps explain certain genotype-phenotype correlations. For example, as discussed in Section. “Genotype-phenotype correlations”, some variants within the CSRD) have been specifically linked to a higher prevalence of learning disabilities and optic pathway gliomas, suggesting a role for this domain beyond Ras regulation in normal neurodevelopment. Consequently, missense variants and small in-frame indels that occur within these key functional regions are often associated with more severe or specific clinical presentations compared to those in less critical areas of the protein
4. **Large deletions:**

This category includes deletions of various sizes with distinct consequences. It is important to distinguish between:

- Whole-gene microdeletions: These are large chromosomal deletions (typically 1.0–1.4 Mb) that remove the entire NF1 gene along with several flanking genes. Often referred to as Type-1 deletions, they account for about 5% of cases [26, 27]. These deletions are consistently associated with a more severe clinical phenotype due to a contiguous gene syndrome, which is discussed further in Section. “Genotype-phenotype correlations”.
- Intragenic (multi- or single-) exon deletions: These are smaller deletions occurring within the NF1 gene itself. Although “large” compared to a single base pair change, their functional consequence is typically a frameshift leading to a truncated protein. As a result, their clinical presentation generally aligns with other truncating variants rather than the severe phenotype seen with whole-gene microdeletions.

In contrast to deletions, whole-gene duplications of the *NF1* region (reciprocal microduplications) result in a completely different clinical presentation. Individuals with these duplications do not have NF1 but instead present with a distinct syndrome characterized by developmental delay, intellectual disability, and a higher prevalence of autism spectrum disorder [28]. Notably, some features are opposite to those seen in NF1, such as microcephaly being more common than macrocephaly. This reciprocal phenotype highlights the critical importance of neurofibromin gene dosage for normal neurodevelopment: too little protein (haploinsufficiency) causes NF1, while too much (triplosensitivity) leads to a different disorder [29].

5. **In-frame deletions/insertions:** These involve the deletion or insertion of one or more amino acids without disrupting the reading frame. A specific three-base pair deletion in exon 17 (c.2970-2972delAAT) is a notable example of a recurrent mutation associated with a milder phenotype, often characterized by the absence of discrete cutaneous neurofibromas.

The vast spectrum of mutations, ranging from those that completely abolish protein function to those that may only partially impair it, provides the initial layer of genetic complexity that underpins the variable expressivity of NF1.

### The role of neurofibromin in cellular signaling

The primary and most well-established function of neurofibromin is to act as a tumor suppressor by negatively regulating the Ras-MAPK pathway (Fig. 1C). Ras proteins are a family of small GTPases that function as molecular switches in signal transduction cascades, controlling fundamental cellular processes such as proliferation, differentiation, survival, and migration. They cycle between an active, GTP-bound state and an inactive, GDP-bound state [30, 31].

Neurofibromin, through its GRD, functions as a Ras-GAP, accelerating the intrinsic GTPase activity of Ras by several orders of magnitude. This action converts active Ras-GTP to inactive Ras-GDP, effectively turning off the signaling pathway [16, 24]. When neurofibromin is absent or non-functional due to *NF1* gene mutations, Ras proteins remain locked in their active GTP-bound state. This leads to constitutive, uncontrolled activation of downstream effector pathways, most notably the extracellular signal-regulated Kinase (Raf-MEK-ERK) mitogen-activated protein kinase (MAPK) pathway [32]. The resulting hyperactivation of this cascade promotes excessive cell growth and proliferation and inhibits apoptosis, which are hallmark characteristics of cancer (Fig. 1C). This mechanism is central to the development of the various tumors seen in NF1, including neurofibromas, optic gliomas, and MPNSTs.

While Ras regulation is its cornerstone function, emerging evidence indicates that neurofibromin’s role in cellular signaling is more complex. It has been shown to interact with and modulate other important pathways. For instance, neurofibromin can influence the cyclic AMP (cAMP)-protein kinase A (PKA) pathway [33]. It appears that cAMP levels can modulate neurofibromin’s ability to bind to Ras [34], suggesting a crosstalk between these two critical signaling networks. Dysregulation of the cAMP pathway in NF1-deficient cells has been implicated in learning and memory deficits observed in animal models and patients [35, 36]. Furthermore, interactions with the mTOR (mammalian target of rapamycin) pathway, another crucial regulator of cell growth and metabolism, have been described [37, 38]. This functional pleiotropy, where neurofibromin participates in multiple signaling networks, helps explain the wide range of tissues affected and the diverse clinical manifestations associated with NF1, extending beyond tumorigenesis to include cognitive, skeletal, and developmental abnormalities. The disruption of this intricate signaling hub by *NF1* pathogenic variant sets the stage for the highly variable clinical outcomes that define the disorder.

## Mechanisms of phenotypic variation

**Fig. 2:**
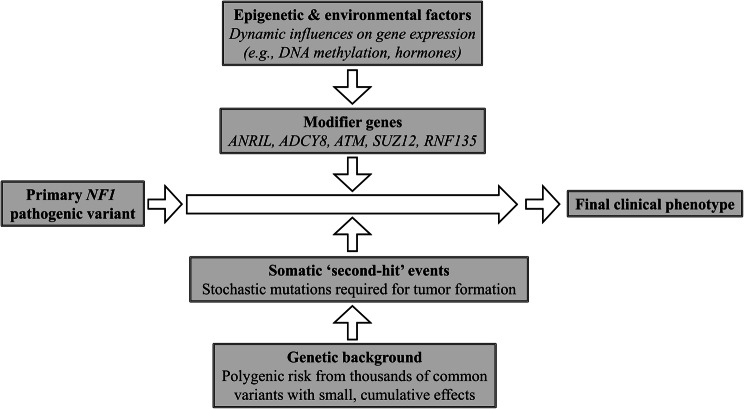
A hierarchical model of phenotypic variability in NF1. The clinical presentation in NF1 results from a multi-layered process. The primary NF1 pathogenic variant establishes the disease baseline. Progression toward the final clinical phenotype is then strongly influenced by several key determinants: modifier genes with significant individual effects on specific traits; stochastic somatic “second-hit” events required for tumorigenesis; the broader genetic background, representing the cumulative polygenic effect of many common variants; and dynamic epigenetic and environmental factors. The unique combination of these influences shapes the final variable clinical outcome for each individual

NF1 serves as a classic example of a single-gene disorder with profound phenotypic variability. While mutations in the *NF1* gene are the necessary cause, they are not sufficient to explain the vast differences in clinical presentation observed among affected individuals. The expressivity of the disease is remarkably heterogeneous, ranging from individuals with only mild dermatological signs to those with severe, life-limiting complications such as malignant tumors, debilitating skeletal abnormalities, and significant neurological impairment. This variability is evident not only between unrelated individuals but also within families sharing the same germline *NF1* pathogenic variant, underscoring the critical role of additional genetic, epigenetic, and environmental factors in shaping the clinical course of the disease. The primary mechanisms contributing to this phenotypic diversity are summarized in Fig. 2. Understanding these modifying mechanisms is paramount for improving prognostic accuracy, developing personalized risk assessments, and designing targeted therapeutic interventions. This section explores the primary mechanisms that contribute to the phenotypic diversity of NF1, including established genotype-phenotype correlations, the influence of modifier genes, the impact of somatic mosaicism and second-hit mutations, and the emerging role of epigenetic and environmental influences.

### Genotype-phenotype correlations

Despite the extensive variability in NF1, research has successfully identified a few specific correlations between the type of *NF1* pathogenic variant (genotype) and certain clinical features (phenotype). These correlations, while accounting for only a fraction of the overall clinical diversity, represent crucial insights into the functional domains of the neurofibromin protein and have direct implications for patient management and genetic counseling. Establishing these links has been challenging due to the large size of the *NF1* gene, the high mutational rate, and the wide array of private mutations. However, large-scale cohort studies have revealed several consistent patterns.

One of the most well-characterized genotype-phenotype correlations involves individuals with a microdeletion of the entire *NF1* gene and its flanking regions on chromosome 17q11.2. These deletions, which account for approximately 5–10% of all NF1 cases, are consistently associated with a more severe clinical phenotype [27]. Individuals with *NF1* microdeletions often present with an earlier onset and a greater number of cutaneous neurofibromas compared to those with intragenic mutations [39]. Furthermore, this group exhibits a significantly higher risk of developing MPNSTs, one of the most serious complications of NF1. Beyond the typical NF1 features, these patients frequently display dysmorphic facial features, developmental delay, intellectual disability, and excessive somatic growth, characteristics not typically associated with intragenic mutations. This more severe presentation is attributed to the contiguous gene syndrome, where the deletion of neighboring genes, in addition to *NF1*, contributes to the complex phenotype [39]. For example, the common 1.4 Mb Type-1 deletion includes the SUZ12 gene, a core component of the PRC2 epigenetic regulatory complex [40]. SUZ12 haploinsufficiency is hypothesized to contribute to the significantly increased risk of MPNSTs in individuals with these microdeletions [41, 42].

Conversely, a specific three-base-pair in-frame deletion in exon 17 (c.2970_2972delAAT) is associated with a milder phenotype, characterized primarily by café-au-lait macules and skinfold freckling, with a notable absence of cutaneous or plexiform neurofibromas and other major complications [43]. This specific mutation provides a valuable tool for genetic counseling, allowing for a more reassuring prognosis for individuals who carry it. This principle is clearly illustrated by a distinct group of missense variants affecting a specific region of the GRD. As established by Koczkowska et al., missense variants in codons 844–848 are associated with a severe, Noonan-like phenotype [44]. Individuals with these variants present with features of Noonan syndrome, such as short stature, pulmonic stenosis, and distinctive facial features, in addition to the classic signs of NF1. This demonstrates that pathogenic variants in certain functionally critical hotspots can lead not only to a loss of canonical neurofibromin function but also to specific and more severe clinical outcomes, likely by uniquely altering the protein’s interaction with the Ras pathway. This observation suggests that these specific regions of neurofibromin play unique roles in developmental pathways shared with other RASopathies.

Beyond these specific examples, broader correlations have been tentatively established synthesizing data from large patient cohorts. For instance, individuals with truncating variants (nonsense or frameshift) that lead to a complete loss of protein function are often thought to have a more severe overall disease burden, including a higher prevalence of optic pathway gliomas and skeletal abnormalities, compared to those with many missense variants that may retain partial protein function [45]. However, this is a general trend with numerous exceptions (such as the severe missense variants in codons 844–848, highlighting the substantial influence of other modifying factors [46]. The location of the variant within the gene can also be significant outside of the major hotspots; for example, pathogenic variants clustering within the CSRD have been linked to a higher prevalence of learning disabilities [47]. These established correlations, though limited, underscore that the nature of the primary *NF1* pathogenic variant itself is a fundamental determinant of the clinical phenotype, providing a baseline upon which other modifying factors exert their influence [48].

### The role of modifier genes

The strong influence of genetic modifiers in NF1 was first clearly demonstrated in a landmark study by Easton et al. in 1993. By analyzing clinical features in a large cohort of related individuals, they showed that disease severity correlated significantly more in first-degree relatives (such as siblings) than in more distant relatives (such as cousins) who shared the same primary NF1 pathogenic variant. This provided strong statistical evidence that genes at other loci, which are shared more among close relatives, modulate the clinical phenotype [6]. Modifier genes are defined as genes at other loci that alter the phenotypic expression of a primary disease-causing mutation. These genes can influence the severity, penetrance, or age of onset of specific NF1-related traits by interacting with the *NF1* gene product or its associated signaling pathways [49–51]. The identification of these modifiers is a key area of research, as it promises to unravel the complex biological networks underlying NF1 pathogenesis and may reveal novel therapeutic targets.

The search for modifier genes has employed various strategies, including candidate gene approaches and genome-wide association studies (GWAS). Candidate gene studies focus on genes known to function in pathways regulated by neurofibromin, such as the Ras/MAPK, cAMP, and mTOR pathways. For example, variations in genes that encode components of these pathways could either exacerbate or ameliorate the effects of neurofibromin haploinsufficiency. If a particular polymorphism in a downstream effector of Ras signaling leads to its hyperactivation, it might synergize with the *NF1* pathogenic variant to promote more aggressive tumor growth. Conversely, a variant that dampens pathway activity could have a protective effect.

GWAS provide an unbiased method for identifying genetic modifiers by scanning the entire genome for common variants associated with specific NF1 phenotypes in large patient cohorts [52, 53]. These studies have begun to yield promising results. For example, a recent large-scale study by Pacot et al. examining cutaneous neurofibroma (cNf) burden identified a significant locus on chromosome 14q. Although the precise causal gene within this region has not yet been definitively validated, candidates include ADCY8, which is involved in the cAMP pathway, a key network regulated by neurofibromin. Another study identified variants in the non-coding RNA ANRIL as associated with the total number of neurofibromas [54]. Similarly, research into the genetic basis of scoliosis in NF1 has shown significant familial aggregation of the trait, providing strong statistical evidence for the influence of genetic modifiers [55]. However, although the heritability of the trait is clearly high, specific unlinked modifier loci for NF1-associated scoliosis have not yet been definitively identified or validated, and this remains an active area of investigation. These findings highlight a critical challenge in modifier gene research: while GWAS can pinpoint associated loci, these regions often contain multiple genes, and extensive functional studies are required to identify the causal variant and clarify its biological impact on the NF1 phenotype.

The genetic background of an individual, composed of thousands of common and rare variants, collectively contributes to their unique susceptibility to different NF1 manifestations [56]. It is unlikely that a single modifier gene explains a large proportion of the variability for any given trait [49, 51]. Instead, it is more plausible that a polygenic model, involving the combined, subtle effects of variants in many different genes, is at play [51, 53]. This polygenic architecture makes the identification of modifiers challenging but also reflects the true complexity of human genetic disease. Understanding this network of genetic interactions is crucial for moving beyond a one-size-fits-all approach to NF1 management and toward a new era of personalized medicine based on an individual’s complete genetic profile.

### Second-hit mutations and somatic mosaicism

**Fig. 3:**
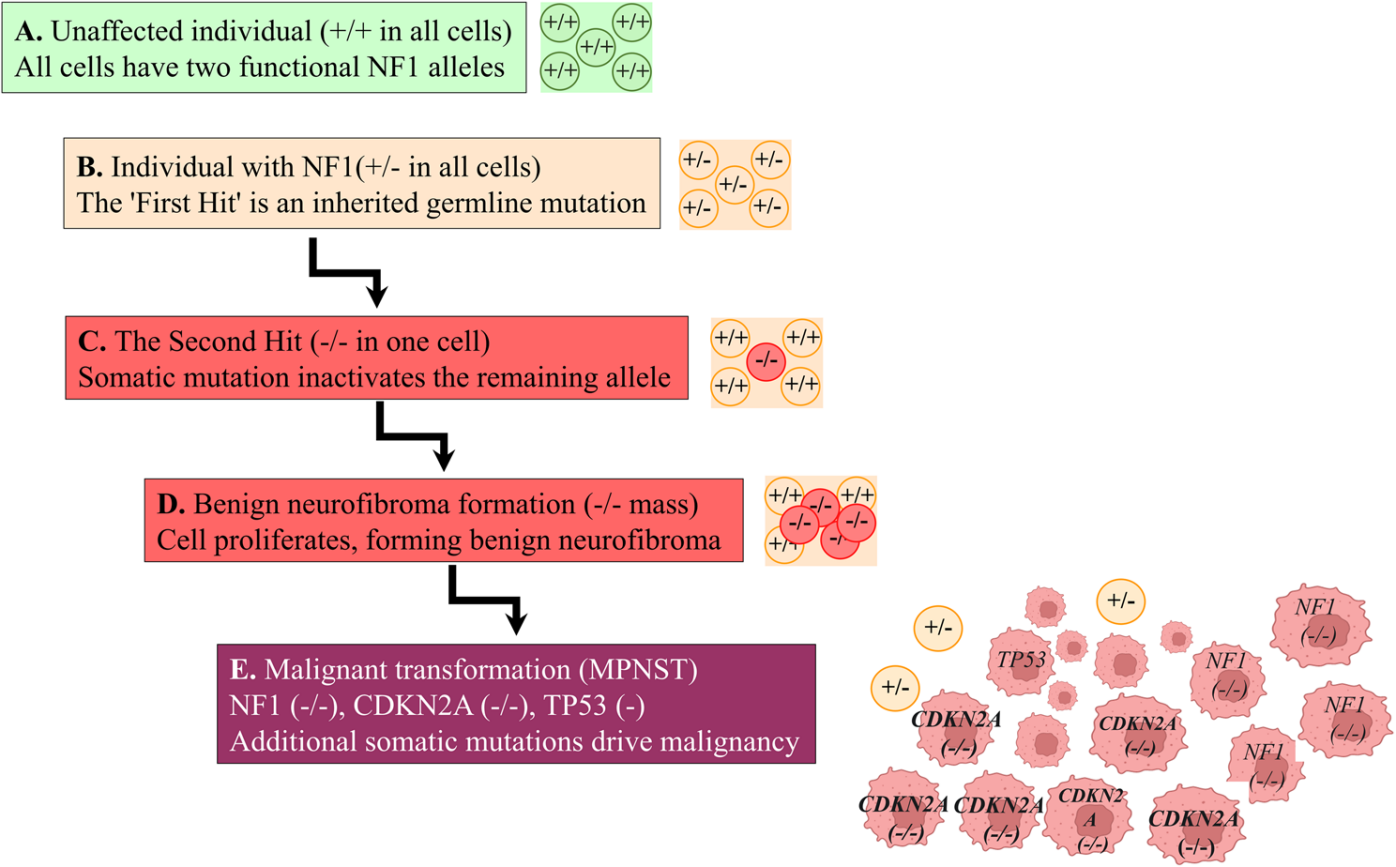
Knudson’s two-hit hypothesis and multistep tumorigenesis in NF1. (**A**) In an unaffected individual, all cells have two functional (+/+) copies of the NF1 gene. (**B**) An individual with NF1 has an inherited germline mutation (the “first hit”), so every cell has only one functional allele (±). The following steps illustrate the timeline of tumorigenesis in this individual’s tissue. (**C**) Tumorigenesis begins when a single cell acquires a somatic mutation (the “second hit”). (**D**) This cell, now with biallelic inactivation of NF1 (-/-), undergoes clonal proliferation to form a benign neurofibroma. (**E**) Progression to a malignant peripheral nerve sheath tumor (MPNST) is driven by the accumulation of additional somatic mutations in other critical genes, such as CDKN2A and TP53

The manifestation of many NF1 features, particularly tumor formation, is contingent upon somatic genetic events that occur after conception. This concept is central to understanding the localized and variable nature of the disease [57]. The “two-hit hypothesis,” originally proposed by Alfred Knudson [58, 59], provides a foundational framework for this process (Fig. 3). In individuals with NF1, the first “hit” is the inherited germline mutation in one copy of the *NF1* gene, which is present in every cell of the body. This leaves a single functional copy of the gene. The second “hit” is a somatic mutation that inactivates the remaining wild-type *NF1* allele in a specific cell, such as a Schwann cell precursor. This complete loss of neurofibromin function in a single cell provides a strong proliferative advantage and is a critical initiating event for tumor formation, most notably for neurofibromas and MPNSTs [60, 61].

The stochastic nature of these second-hit mutations helps to explain the variability in the number, location, and timing of tumor development among individuals with the same germline mutation. The probability of a somatic mutation occurring is not uniform across all tissues or over a person’s lifetime, leading to a mosaic pattern of cellular genotypes within the body [62]. Factors such as exposure to mutagens, errors in DNA replication, and the intrinsic mutation rate of a specific cell type can all influence the likelihood of a second hit. This explains why an individual might develop hundreds of neurofibromas while a sibling with the identical germline mutation develops only a few.

The concept of mosaicism also applies to the primary NF1 pathogenic variant, which can arise post-zygotically during embryonic development [53, 63, 64]. This results in two distinct forms with important implications for genetic counseling. Somatic mosaicism produces a segmental form of NF1, in which clinical features such as café-au-lait macules and neurofibromas are limited to a specific area of the body, reflecting the distribution of the mutated cell lineage. These individuals are often more mildly affected and may not meet the full diagnostic criteria. In contrast, germline mosaicism occurs when the mutation is present in a proportion of the germline cells (sperm or oocytes). An individual with germline mosaicism may have few or no clinical features of NF1 but can have children with classic, non-mosaic NF1 because they can transmit the mutation [53, 65]. Recognizing both possibilities is essential for accurate diagnosis and counseling

The mechanism of the second hit itself can also influence the phenotype. The complete loss of the wild-type allele, often through a large deletion or mitotic recombination, is a common event in the development of benign neurofibromas. However, the progression from a benign plexiform neurofibroma to a malignant MPNST involves the accumulation of additional somatic mutations in other cancer-related genes, such as *TP53* and *CDKN2A*. This multi-step process of tumorigenesis, initiated by the loss of *NF1* and driven by subsequent genetic alterations, highlights how somatic events are a major driver of phenotypic variability and disease severity, particularly concerning the risk of malignancy [66, 67].

### Epigenetic and environmental factors

While genetic factors like the primary mutation, modifier genes, and somatic events are fundamental drivers of phenotypic variability, they do not operate in a vacuum. Epigenetic modifications and environmental exposures add another layer of complexity, influencing how the genetic blueprint is expressed. Epigenetics refers to heritable changes in gene expression that occur without altering the underlying DNA sequence. These mechanisms, including DNA methylation, histone modification, and non-coding RNAs, can be dynamic and are responsive to environmental cues, providing a potential link between external factors and disease expression.

DNA methylation is one of the most studied epigenetic marks. Hypermethylation of the promoter region of a gene can lead to its transcriptional silencing. In the context of NF1, hypermethylation of the promoter of the wild-type *NF1* allele has been identified as a mechanism for the “second hit” in some tumors [68, 69]. This epigenetic silencing achieves the same functional outcome as a somatic mutation, the complete loss of neurofibromin expression, thereby initiating tumorigenesis. The frequency and pattern of such epigenetic events could be influenced by both genetic predisposition and environmental factors, contributing to the variability in tumor burden.

Histone modifications, which influence chromatin structure and function, are also critical. For example, a key epigenetic event in the malignant transformation of neurofibromas is the global loss of histone H3 lysine 27 trimethylation (H3K27me3). This repressive mark is lost due to inactivating mutations in components of the Polycomb Repressive Complex 2 (PRC2), such as EED or SUZ12, a mechanism central to MPNST development that will be discussed further [41]. This demonstrates a direct link in which a specific epigenetic alteration, driven by somatic mutation, profoundly changes the tumor phenotype.

Non-coding RNAs, such as microRNAs (miRNAs), add an additional layer of post-transcriptional regulation. MiRNAs are small RNA molecules that bind to messenger RNA (mRNA), leading to its degradation or translational repression. In NF1, several miRNAs are dysregulated. For example, miR-10b is overexpressed in MPNSTs, where it promotes tumor cell invasion and metastasis by targeting genes involved in cell adhesion [70]. Other miRNAs may directly or indirectly affect the Ras pathway, acting as oncogenes or tumor suppressors [71]. Individual variability in the expression of these and other non-coding RNAs may therefore contribute significantly to phenotypic heterogeneity.

While epigenetic marks provide the molecular machinery for variable gene expression, environmental factors are strong candidates for triggering these changes over a person’s lifetime. Although this area of NF1 research is still developing, several plausible environmental influences have already been proposed. The most clinically well-known example is the influence of hormonal changes [72]; many patients report an increase in the number and size of cutaneous neurofibromas during puberty and pregnancy, times of significant steroid hormone fluctuations. This suggests that hormones associated with NF1 haploinsufficiency can strongly modulate cell proliferation.

It is assumed that other factors based on cancer biology principles also play a role. Chronic inflammation, for example, is an important candidate. Neurofibromas are known to be rich in mast cells and other immune infiltrates, creating a pro-inflammatory microenvironment that can promote cell growth and survival [62]. It is plausible that systemic inflammatory conditions influenced by diet or chronic disease could exacerbate this effect. Similarly, exposure to mutagens, such as ultraviolet (UV) radiation, could theoretically increase the rate of somatic “second-hit” mutations in Schwann cell precursors in the skin. These environmental influences could have an effect by triggering somatic mutations or altering the epigenetic state of the cells, thereby modulating signaling pathways that interact with neurofibromin [69].

The investigation of these environmental relationships posed a major challenge. First, NF1 is a rare disease, making it difficult to assemble the large patient cohorts needed for statistically meaningful epidemiologic studies. Second, quantifying cumulative lifetime exposure to diffuse factors such as diet or inflammation is notoriously difficult and often relies on patient recall, which can be unreliable. Finally, clinical variability in NF1 is already driven by strong genetic determinants (primary mutation, modifier genes) and stochastic events (second-hit mutations), making the subtle, modulating effects of environmental factors difficult to isolate and recognize.

The definitive identification of these environmental factors requires large-scale, longitudinal, international cohort studies. Such studies would need to prospectively follow thousands of individuals with NF1 from an early age, combining thorough genetic and clinical phenotyping with rigorous and repeated collection of environmental data through validated questionnaires, geographic mapping and biological markers of exposure. This multi-omic approach, integrating genomic, epigenomic and exposomic data, is key to definitively elucidating how an individual’s environment interacts with their genetic blueprint and influences the clinical course of NF1.

## Clinical manifestations and genetic links

NF1 is a multisystem disorder with a broad spectrum of clinical manifestations that evolve over an individual’s lifetime. The diagnosis is typically made based on a set of well-defined clinical criteria, which encompass the most common features of the disease. While the expression of these features is highly variable, research has begun to uncover specific genetic links that help explain why certain manifestations occur and how they might be related to the underlying molecular pathology. This part of the review describes the major clinical phenotypes of NF1, linking each manifestation directly to the underlying genetic mechanisms discussed earlier. We show how distinct phenomena, such as haploinsufficiency from the germline mutation, somatic second-hit events, and the influence of modifier genes, produce the specific cutaneous, neurological, skeletal, and neoplastic features of the disorder. This section also examines the multistep genetic process of malignant transformation, the ultimate consequence of these cumulative events. The relationships between these major clinical features and their underlying genetic determinants are summarized in Table 1.

**Table 1:** Genetic mechanisms underlying key clinical manifestations of NF1

Clinical manifestation	Primary genetic mechanism	Known genotype-phenotype correlations	Influence of other factors	References
Café-au-lait Macules	Haploinsufficiency (±)	Present in nearly all genotypes.	Modifier genes influence number and size.	[51]
Cutaneous neurofibromas	Second Hit (-/-)	Absent in c.2970-2972delAAT variant. Increased number in microdeletions.	Strong influence of modifier genes on tumor burden. Hormonal influences (puberty, pregnancy).	[38, 42, 52, 71]
Plexiform neurofibromas	Second Hit (-/-)	Often severe in microdeletions.	Influence of the TME is critical for growth.	[25, 72]
MPNST	Multi-step Hits (-/- + others)	Significantly higher risk in microdeletions (e.g., with *SUZ12* loss).	Accumulation of somatic variants in *CDKN2A*, *TP53*, PRC2 complex is required.	[38, 40, 41, 73, 74]
Cognitive deficits/LD	Haploinsufficiency (±)	Can be more severe in microdeletions. Higher prevalence with CSRD variants.	Influence of modifier genes and genetic background is likely high.	[38, 46]
Optic pathway gliomas	Second Hit (-/-)	Increased prevalence with variants in the 5’ region of the gene.	TME plays a key role in tumor development.	[75, 76]
Skeletal dysplasia	Haploinsufficiency (±)	Specific skeletal issues can be part of certain phenotypes (e.g., Noonan-like).	High variability suggests strong influence of modifier genes (active area of research).	[6, 43, 54]
Lisch nodules	Haploinsufficiency (±)	Present in >90% of adults across most genotypes.	-	[77, 78]

NF1: Neurofibromatosis Type 1, MPNST: malignant peripheral nerve sheath tumors, CSRD: cysteine-serine-rich domain,TME: Tumor microenvironment

### Cutaneous and neurological phenotypes

The most visible and common manifestations of NF1 are found in the skin and nervous system. Café-au-lait macules are the hallmark feature, appearing in the first year of life in nearly all affected individuals. These hyperpigmented skin lesions are a direct result of *NF1* haploinsufficiency in melanocytes, leading to increased melanin production. While their number and size are part of the diagnostic criteria, they are not typically associated with other disease complications. Skinfold freckling, appearing in the axillary or inguinal regions, shares a similar pathogenesis.

Neurofibromas are the characteristic tumors of NF1 and the primary cause of morbidity associated with the disorder. They are benign peripheral nerve sheath tumors composed of a heterogeneous mix of cell types, including Schwann cells, fibroblasts, perineurial cells, and mast cells.

Although the biallelic inactivation of NF1 in the precursors of Schwann cells is the decisive triggering event, these tumors are not simply monoclonal expansions. Rather, they develop into a complex cellular ecosystem in which the tumor microenvironment (TME) plays a crucial role in their growth and persistence. The NF1 -/- Schwann cells recruit and interact with a variety of other cell types. Mast cells, for example, are abundant in neurofibromas and create a pro-inflammatory and angiogenic environment by releasing factors such as histamine, proteases and cytokines. This inflammatory environment is thought to be an important factor in tumorigenesis. Fibroblasts contribute by depositing extracellular matrix and secreting growth factors that promote the proliferation of neoplastic Schwann cells [79]. This intricate interplay within the TME helps to explain the clinical variability of tumor burden. Differences in the composition and activity of the microenvironment in different individuals may contribute to some patients developing thousands of neurofibromas while others develop far fewer [72]. This biological complexity presents both a therapeutic challenge, since targeting only the neoplastic Schwann cells may be insufficient, and an opportunity, as the non-neoplastic cells of the TME offer alternative targets for intervention, as discussed in Section. “Targeted therapies and future prospects”

The development of neurofibromas is contingent upon a “second hit” that inactivates the wild-type *NF1* allele in a Schwann cell precursor (Fig. 3D) [57, 62]. There are several distinct types of neurofibromas:

**• Cutaneous neurofibromas** are soft, fleshy growths on or under the skin. They typically appear during puberty or early adulthood, and their number can range from a few to thousands, increasing with age. The variability in the number of cutaneous neurofibromas is a classic example of the influence of modifier genes, as individuals with identical germline mutations can have vastly different tumor burdens [56].

**• Subcutaneous neurofibromas** are firmer, more discrete nodules located along peripheral nerves deeper under the skin.

**• Plexiform neurofibromas** are more extensive tumors that grow along the length of nerves and can involve multiple nerve fascicles. They are often congenital and can cause significant disfigurement, pain, and functional impairment by compressing adjacent structures. Approximately 30–50% of individuals with NF1 develop plexiform neurofibromas. While they are benign, they carry a lifetime risk of 8–13% of transforming into MPNST [75].

Beyond peripheral tumors, NF1 has significant neurological implications. Optic pathway gliomas (OPGs) are low-grade tumors that occur in approximately 15–20% of children with NF1, typically before the age of six [76, 80]. These tumors arise from the complete loss of *NF1* in glial cells of the optic nerve. While many OPGs are asymptomatic and remain stable, some can cause progressive vision loss and other neurological deficits, requiring therapeutic intervention [81, 82]. Certain germline mutation locations, particularly in the 5’ region of the *NF1* gene, have been associated with an increased risk of developing OPGs.

Cognitive and learning disabilities are another major neurological component of NF1, affecting up to 80% of children with the condition [83]. These deficits can manifest as specific learning disorders (e.g., in reading or math), attention-deficit/hyperactivity disorder (ADHD), and challenges with executive function and visuospatial skills [84]. Unlike tumors, these cognitive issues are not caused by second-hit mutations but are thought to result from *NF1* haploinsufficiency in the central nervous system. This distinction is critical, as it highlights how different mechanisms drive distinct aspects of the NF1 phenotype: biallelic inactivation (-/-) is required for tumorigenesis, whereas haploinsufficiency (±) is sufficient to disrupt the development and function of the central nervous system. Neurofibromin plays a critical role in regulating synaptic plasticity and neurotransmitter release, and its deficiency disrupts normal neuronal function and brain development [85]. The severity of cognitive impairment is highly variable and appears to be influenced by both the specific *NF1* pathogenic variant and the broader genetic background of the individual.

### Skeletal and ocular abnormalities

Skeletal abnormalities are common in NF1, affecting bone growth and development. These manifestations are believed to stem from the dysfunctional regulation of bone-forming cells (osteoblasts) and bone-resorbing cells (osteoclasts) due to *NF1* haploinsufficiency [86, 87]. One of the most characteristic skeletal features is tibial dysplasia, or bowing of the long bones of the lower leg, which can be present at birth and may lead to fracture and the formation of a pseudarthrosis (a “false joint”) that is difficult to heal [88]. Sphenoid wing dysplasia, an abnormality of the bone behind the eye, is another specific diagnostic criterion [89].

Scoliosis, or curvature of the spine, is the most frequent skeletal problem in NF1, affecting 10–30% of individuals. It often presents as a short, sharp curve (dystrophic scoliosis) that can be progressive and may require surgical correction [77, 90]. The genetic basis for the variable penetrance of these skeletal defects is an active area of investigation, with genome-wide studies seeking to identify modifier genes that predispose individuals to more severe bone complications.

The ocular system is also frequently affected in NF1. Lisch nodules are benign hamartomas (small, pigmented growths) on the surface of the iris. They are present in over 90% of adults with NF1 and are a valuable diagnostic sign, though they do not cause any visual symptoms [78, 91]. Their presence is a direct consequence of *NF1* haploinsufficiency. As previously mentioned, optic pathway gliomas represent a more serious ocular complication that can directly threaten vision. The combined presence of Lisch nodules and the risk of OPGs necessitates regular ophthalmological examinations for all individuals with NF1, particularly in childhood. The distinct genetic bases of these two ocular features provide a clear example of NF1 pathogenesis. Lisch nodules result directly from haploinsufficiency in the iris, while OPGs require a second hit to initiate tumorigenesis in the optic nerve. This demonstrates how different mechanisms within the same organ system can lead to very different clinical outcomes, one benign and the other potentially sight-threatening.

### Malignant transformation and tumorigenesis

While the majority of tumors in NF1 are benign, the lifetime risk of developing a malignancy is significantly increased compared to the general population [64, 67]. The most common malignancy associated with NF1 is the MPNST, an aggressive sarcoma that typically arises from pre-existing plexiform neurofibromas. The lifetime risk of developing an MPNST for an individual with NF1 is estimated to be 8–13% [73, 92].

The genetic pathway leading to MPNST is a multi-step process that begins with the biallelic inactivation of the *NF1* gene in a Schwann cell, leading to the formation of a neurofibroma [41]. The progression from a benign neurofibroma to an atypical neurofibroma and finally to an MPNST is driven by the acquisition of additional somatic mutations in other critical tumor suppressor genes and oncogenes. The most common secondary genetic event in MPNST development is the inactivation of the *CDKN2A* tumor suppressor gene, which regulates the cell cycle [41]. This is often followed by mutations in other genes involved in cell growth and survival pathways, such as the Polycomb Repressive Complex 2 (PRC2) components *EED* or *SUZ12*. Mutations in the *TP53* tumor suppressor gene are also frequently found in high-grade MPNSTs and are associated with a poorer prognosis (Fig. 3E) [74, 93].

This model of multi-step tumorigenesis explains why only a subset of plexiform neurofibromas undergo malignant transformation. The probability of acquiring the necessary constellation of secondary mutations is a stochastic process. However, specific germline *NF1* genotypes appear to modify this risk. As noted, individuals with large *NF1* microdeletions have a demonstrably higher risk of developing MPNSTs, suggesting that the loss of neighboring genes may create a cellular environment more permissive for malignant transformation [42, 67].

Beyond MPNSTs, individuals with NF1 have an increased risk of other malignancies, including juvenile myelomonocytic leukemia (JMML) [94, 95], a rare childhood leukemia strongly associated with *NF1* pathogenic variants; pheochromocytomas (tumors of the adrenal gland) [96, 97]; gastrointestinal stromal tumors (GISTs) [98]; and breast cancer [99, 100]. In each of these cases, the *NF1* pathogenic variant acts as a potent tumor-predisposing factor, and the development of cancer relies on the accumulation of additional tissue-specific somatic genetic and epigenetic alterations [101]. Understanding the precise molecular pathways that drive malignant transformation in different cellular contexts is a central goal of NF1 research, as it is essential for developing effective surveillance strategies and targeted therapies to combat these life-threatening complications [102, 103].

## Diagnostic and therapeutic implications

The profound phenotypic variability in NF1 presents significant challenges for diagnosis, prognostication, and management. However, advancing knowledge of the genetic and molecular underpinnings of this variability is paving the way for more precise diagnostic tools, personalized counseling, and innovative therapeutic strategies. This section explores the clinical applications of our evolving understanding of NF1 genetics, focusing on genetic testing, counseling, and the development of targeted therapies.

### Advances in genetic testing and counseling

The cornerstone of NF1 diagnosis remains clinical, based on the diagnostic criteria established by the NIH [2]. However, genetic testing plays an increasingly vital role, particularly in cases where the clinical presentation is ambiguous, for prenatal or preimplantation genetic diagnosis, and for refining prognostic assessments based on emerging genotype-phenotype correlations [64, 104]. The complexity and size of the *NF1* gene, which spans over 350 kb and contains 60 exons, historically made comprehensive mutation analysis challenging. Modern next-generation sequencing (NGS) techniques have revolutionized this process, enabling high-throughput, cost-effective screening that can detect a wide array of variants, including single nucleotide variants, small indels, and splice-site alterations [105–107].

To capture the full spectrum of NF1 variants, which includes large genomic deletions or duplications in 5–10% of cases, specific copy number variation (CNV) analysis is essential. Multiplex Ligation-dependent Probe Amplification (MLPA) is often used as a first-line screening tool alongside sequencing. Commercial MLPA kits for NF1 are widely available and typically contain probes for all NF1 exons as well as for key flanking genes, such as SUZ12, allowing for the effective detection of the common Type-1 microdeletion [108, 109]. However, as MLPA is a targeted assay, it may not identify deletions with atypical breakpoints. Therefore, for cases where a large deletion is suspected but not detected by MLPA, or for a more comprehensive and unbiased analysis, chromosomal microarray analysis (CMA) is the recommended methodology. CMA, which includes array comparative genomic hybridization (aCGH) and SNP arrays, can accurately define the size and gene content of both typical and atypical CNVs across the 17q11.2 region and genome-wide. Following CNV detection, RNA-based analyses can be employed to identify deep intronic variants that lead to splicing defects, which may be missed by conventional DNA sequencing alone. The comprehensive identification of the underlying pathogenic variant in an individual is the first step toward personalized medicine. It confirms the diagnosis in equivocal cases and allows for cascade testing within families, which is crucial given the 50% chance of transmission to offspring [110–112].

The identification of specific *NF1* pathogenic variants informs genetic counseling by providing more tailored prognostic information. For instance, individuals with a whole-gene deletion are known to be at a higher risk for developing MPNSTs, cognitive impairment, and dysmorphic features [42, 67, 113]. Conversely, patients with the c.2970-2972delAAT (p.Met992del) pathogenic variant typically present with a milder phenotype characterized by pigmentary changes but a notable absence of discrete neurofibromas [43, 114]. Knowledge of missense mutations affecting codons 844–848 has been linked to a “Noonan-like” phenotype, including pulmonic stenosis and distinct facial characteristics, without the development of cutaneous neurofibromas [44, 45]. This ability to link genotype to potential clinical outcomes, while still imperfect, empowers clinicians and families to engage in more informed discussions about disease surveillance, risk management, and life planning. It transforms genetic counseling from a conversation about general probabilities to one that incorporates personalized risk stratification, allowing for anticipatory guidance tailored to the specific genetic context of the patient [115].

**Fig. 4:**
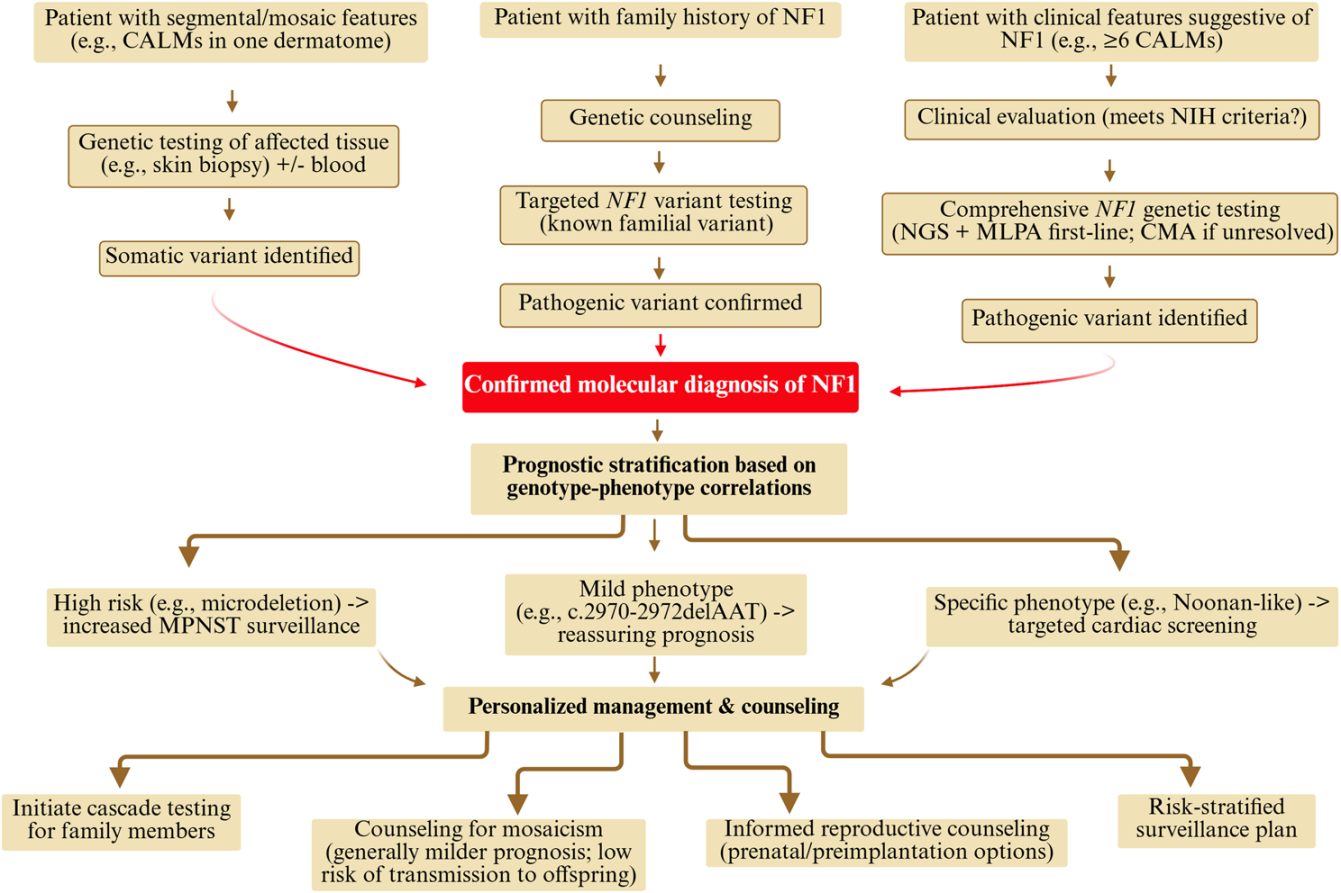
A modern clinical workflow for the diagnosis and management of NF1. The integration of genetic knowledge has transformed the clinical pathway for NF1, accommodating various presentations, including familial, sporadic (suspected constitutional), and mosaic cases. A stepwise genetic testing approach is typically used, with NGS and MLPA as first-line tools and CMA reserved for more complex cases. Once a pathogenic variant is identified, this molecular information is used for prognostic stratification to tailor surveillance, such as increased MPNST monitoring for high-risk genotypes. This enables personalized management, including cascade testing for at-risk relatives and providing informed genetic counseling specific to the individual’s diagnosis (constitutional or mosaic), ultimately moving beyond a uniform model to precision medicine

The practical impact of these advances on clinical practice is significant, creating a more genetics-led diagnostic and management workflow (Fig. 4). For individuals with a suspected diagnosis but an ambiguous clinical presentation, genetic testing can provide a definitive molecular diagnosis. Once a pathogenic variant is identified, it unlocks the power of prognostic stratification. For instance, as discussed, identifying a microdeletion alerts the clinical team to a higher lifetime risk of MPNSTs, prompting more intensive surveillance. Conversely, identifying a variant like c.2970-2972delAAT allows for a more reassuring prognosis regarding the risk of neurofibromas. This molecular information is also the cornerstone of preventative family medicine, enabling efficient cascade testing for at-risk relatives and providing families with concrete information for reproductive counseling, including options for prenatal or preimplantation genetic testing. This structured approach demonstrates how our understanding of genetic determinants is directly translating into more precise, personalized, and proactive clinical care.

### Targeted therapies and future prospects

**Fig. 5:**
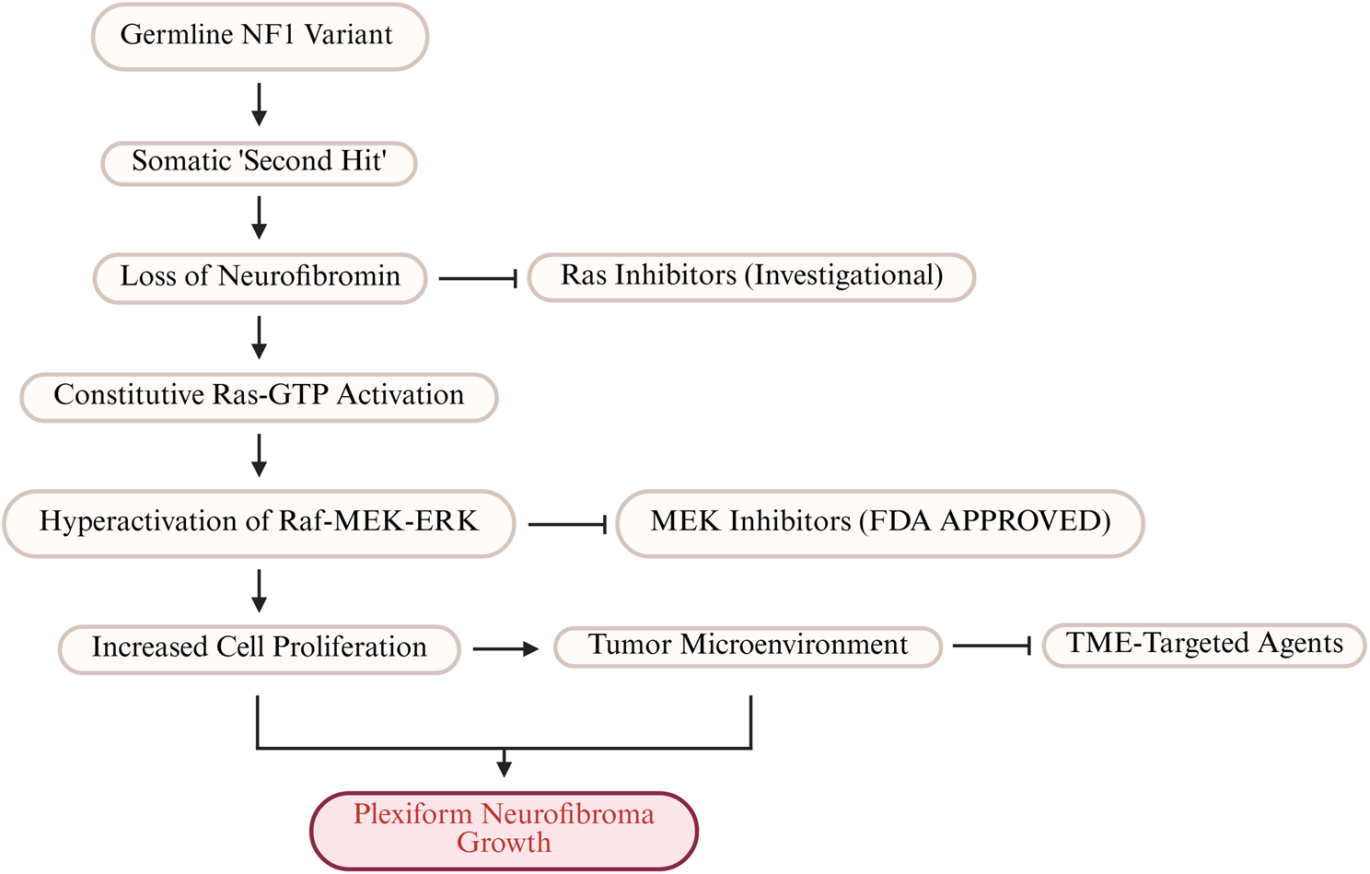
The pathogenesis of neurofibroma formation and key therapeutic targets. Tumorigenesis in NF1 is a multistep process, creating several opportunities for therapeutic intervention. After the “first hit” (germline variant) and “second hit” (somatic variant), loss of functional neurofibromin leads to constitutive Ras-GTP activation and subsequent hyperactivation of the Raf-MEK-ERK signaling cascade. This drives abnormal cell proliferation and survival, and promotes a pro-tumorigenic microenvironment rich in mast cells and inflammation, all contributing to plexiform neurofibroma (PN) growth. Key therapeutic strategies, indicated by inhibitory arrows, are designed to inhibit specific nodes in this pathway. MEK inhibitors are clinically approved and have demonstrated success in shrinking PNs. Other approaches, such as direct Ras inhibitors and agents targeting the tumor microenvironment, are under active investigation

The central role of neurofibromin as a negative regulator of the Ras/MAPK signaling pathway has provided a clear molecular target for therapeutic intervention [30]. Dysregulation of this pathway is a key driver of tumorigenesis in NF1, particularly in the formation of plexiform neurofibromas peripheral nerves (PNs) and MPNSTs. This pathogenic cascade, along with key points of therapeutic intervention, is illustrated in Fig. 5. This understanding has led to the development of MEK inhibitors, which target downstream components of the Ras pathway. Selumetinib, a MEK1/2 inhibitor, was the first drug to receive FDA approval for the treatment of pediatric patients with inoperable, symptomatic PNs [116, 117]. Clinical trials demonstrated that selumetinib could produce sustained tumor volume reduction and alleviate associated clinical symptoms, such as pain and disfigurement, marking a significant milestone in NF1 therapeutics [118–120]. This success has spurred the investigation of other MEK inhibitors and combination therapies aimed at providing more effective and durable responses [121, 122]. Despite this landmark success, the use of MEK inhibitors presents several challenges. Patient responses are heterogeneous; while many individuals experience stabilization of their plexiform neurofibromas and improvement in symptoms, only a subset achieves the greater than 20% volume reduction that defines a formal partial response. These agents are also associated with a notable spectrum of side effects, most commonly dermatologic (such as acneiform rash and paronychia), as well as gastrointestinal and cardiac toxicities, which can affect quality of life and may require dose reductions or treatment discontinuation [118, 120]. The potential for acquired resistance, a well-documented phenomenon with targeted therapies in oncology, remains a long-term concern and is an active area of investigation [123].

Beyond MEK inhibition, the therapeutic landscape is expanding to address other consequences of *NF1* pathogenic variant. For instance, since neurofibromin also influences cAMP levels, strategies to modulate this pathway are being explored. An in vitro study studies have shown that treatments increasing intracellular cAMP can inhibit the growth of NF1-deficient cells [34]. Additionally, the complex tumor microenvironment, rich in mast cells and other immune infiltrates, presents another therapeutic avenue. Agents targeting mast cell degranulation or other inflammatory components are under investigation for their potential to slow neurofibroma growth [79, 124].

Future therapeutic prospects will likely involve a multi-pronged approach tailored to the specific genetic and clinical context of the individual. As research on modifier genes progresses, it may become possible to develop therapies that counteract the effects of specific risk-conferring genetic variants. For example, if a modifier gene variant is found to enhance angiogenesis in neurofibromas, patients carrying that variant might be prioritized for anti-angiogenic therapies. Similarly, the identification of epigenetic marks associated with disease severity could lead to the development of “epigenetic drugs” that aim to reverse pathological gene expression patterns.

The ultimate goal is to move from managing complications as they arise to preventing them altogether. This will require not only deeper insights into the molecular drivers of each specific NF1 manifestation but also the development of robust biomarkers that can predict disease course and treatment response. Integrating genomic data with clinical information and longitudinal monitoring will be essential for realizing the promise of precision medicine for individuals with NF1. The journey from identifying the *NF1* gene to approving the first targeted therapy has been long, but it has laid a strong foundation for a future where therapeutic interventions are increasingly personalized and effective [125].

## Outstanding questions and future directions

Looking ahead, while significant progress has been made, several major challenges and outstanding questions remain. The journey to true precision medicine for NF1 will require a concerted effort to address these complex issues. This section outlines the pragmatic next steps that will likely define the future of NF1 research.

### Deciphering the polygenic architecture of modifier genes

A primary challenge is the systematic identification of the full complement of genetic modifiers. Although GWAS have identified several loci [53], a large portion of the heritable variability remains unexplained. It is now widely accepted that most NF1-related traits are influenced by a polygenic architecture, in which hundreds or thousands of variants, each with a very small effect, collectively contribute to the phenotype. Future studies will therefore require (1) even larger, more diverse international cohorts to achieve the statistical power needed to detect these small effects; (2) the application of whole-genome sequencing to move beyond common variants and identify the role of rare variants; and (3) the use of high-throughput functional validation using technologies such as CRISPR screening in relevant cell models to move from statistical association to a causally implicated gene and pathways. The ultimate aim is not just to catalog genes, but to identify novel biological pathways that can be targeted therapeutically.

### Elucidating gene-environment interactions

The role of environmental factors remains one of the least understood areas of NF1 pathogenesis, primarily due to significant methodological hurdles. Quantifying cumulative, low-dose environmental exposures, such as diet and inflammation, over a patient’s lifetime is notoriously difficult, especially in a rare disease where assembling large epidemiological cohorts is challenging. Progress in this area will depend on large-scale, longitudinal prospective studies that enroll patients early in life. These studies must integrate comprehensive genetic and clinical phenotyping with modern “exposomic” techniques, such as using biomarkers in blood or tissue to obtain objective measures of exposure rather than relying on patient recall. Initial research should focus on high-probability candidates, such as rigorously studying the impact of hormonal fluctuations during puberty and pregnancy on neurofibroma growth, to establish a proven paradigm for gene-environment interaction in NF1.

### Integrating multi-omic data for clinical prediction

The future of personalized prognostication in NF1 lies in the ability to integrate large, diverse datasets. An individual’s primary NF1 variant alone is insufficient to predict their clinical course. A truly accurate predictive model must incorporate genomics (modifier genes, genetic background), epigenomics (DNA methylation patterns), transcriptomics (gene expression), and longitudinal clinical data. The development of machine learning and artificial intelligence models is essential to identify complex patterns within these multi-omic datasets. This requires (1) standardized data collection and sharing across international consortia, and (2) rigorous clinical validation of any developed predictive algorithms in independent patient cohorts to ensure their accuracy and reliability for clinical use. The ultimate goal is to develop clinically validated “risk scores” that can predict an individual’s likelihood of developing specific severe complications, such as MPNST or severe scoliosis, thereby enabling personalized surveillance and management strategies [48].

## Conclusion

NF1, once viewed as an inscrutable and unpredictably variable monogenic disorder, is steadily yielding its complexities to systematic genetic and molecular investigation. The extensive phenotypic heterogeneity, ranging from mild dermatological signs to life-threatening malignancies, is no longer seen as random but as the result of a complex interplay of determinants. This paper has synthesized the current understanding of these factors, highlighting how the nature of the primary *NF1* germline pathogenic variant, the influence of genetic modifiers, the impact of somatic “second-hit” events, and the potential roles of epigenetic and environmental factors collectively shape the clinical trajectory of an individual with NF1.

The foundational molecular mechanism driving NF1 pathogenesis is the loss of neurofibromin, a critical negative regulator of the Ras/MAPK pathway. However, the specific type and location of the *NF1* pathogenic variant provide the first layer of predictive information, with clear genotype-phenotype correlations now established for several recurrent variants and whole-gene deletions. These correlations, while powerful, do not fully account for the observed variability, particularly the striking differences seen even among family members sharing the same germline pathogenic variant. This intrafamilial variability underscores the crucial contribution of other genetic and non-genetic factors.

The search for modifier genes has begun to identify loci that modulate the risk and severity of specific NF1-related complications, such as neurofibroma burden and cognitive deficits. These modifiers likely function by altering the Ras signaling pathway or parallel cellular processes, thereby buffering or exacerbating the effects of neurofibromin deficiency. Concurrently, the Knudson two-hit hypothesis remains central to understanding tumorigenesis, where somatic mutations in the wild-type *NF1* allele trigger localized tumor formation. The stochastic nature of these secondary events is a major driver of variability in the number, location, and timing of tumor development. Emerging evidence also points to the involvement of epigenetic modifications and environmental exposures in fine-tuning gene expression and cellular behavior, adding another layer of regulatory complexity.

This multi-faceted understanding has profound diagnostic and therapeutic implications. Advanced genetic testing now enables precise pathogenic variant identification, facilitating more accurate diagnosis and personalized genetic counseling based on known genotype-phenotype relationships. Most importantly, the elucidation of the central role of Ras pathway hyperactivation has culminated in the development of targeted molecular therapies, such as MEK inhibitors, which represent a paradigm shift from supportive care to mechanism-based treatment.

Looking ahead, several major challenges remain. Systematically identifying the full set of genetic modifiers is a formidable task, as most traits are likely influenced by a polygenic architecture involving numerous variants with small individual effects, which requires increasingly large patient cohorts for detection. Delineating the precise mechanisms of epigenetic and environmental influences is an even greater hurdle, given the methodological difficulty of accurately quantifying lifelong environmental exposures in a rare disease population. The ultimate challenge is to integrate multi-omic data – genomics, epigenomics, and proteomics – into robust, predictive models that are clinically useful. Overcoming these obstacles will require continued international collaboration, the development of novel analytical strategies, and deep, longitudinal clinical phenotyping. By addressing these key unanswered questions, the scientific and medical communities can move closer to a future in which every individual with NF1 receives a truly personalized prognosis and tailored management plan, paving the way for an era of genuine precision medicine for this complex disorder.

## Data Availability

No new data were generated during the preparation of this review. All information reported in this manuscript was extracted from publicly available scientific literature.

## Abbreviations

NF1: Neurofibromatosis Type 1
NIH: National Institutes of Health
MPNSTs: Malignant peripheral nerve sheath tumors
Kb: Kilobases
mRNA: Messenger RNA
NTD: N-terminal domain
GAP: GTPase-activating protein
GAPex: GAP-extra domain
Sec14-PH: Secretory protein 14-pleckstrin homology-like module
CDM: Central dimerization module
CTD: C-terminal domain
CSRD: Cysteine-serine-rich domain
MAPK: Mitogen-activated protein kinase
Raf-MEK-ERK: Extracellular signal-regulated Kinase
mTOR: Mammalian target of rapamycin
GWAS: Genome-wide association studies
UV: Ultraviolet
TME: Tumor microenvironment
OPGs: Optic pathway gliomas
ADHD: Attention-deficit/hyperactivity disorder
PRC2: Polycomb Repressive Complex 2
JMML: Juvenile myelomonocytic leukemia
GISTs: Gastrointestinal stromal tumors
NGS: Next-generation sequencing
MLPA: Multiplex Ligation-dependent Probe Amplification
PNs: Peripheral nerves
CMA: Chromosomal microarray analysis
